# Exploring Feasibility of Multivariate Deep Learning Models in Predicting COVID-19 Epidemic

**DOI:** 10.3389/fpubh.2021.661615

**Published:** 2021-07-05

**Authors:** Shi Chen, Rajib Paul, Daniel Janies, Keith Murphy, Tinghao Feng, Jean-Claude Thill

**Affiliations:** ^1^Department of Public Health Sciences, University of North Carolina at Charlotte, Charlotte, NC, United States; ^2^School of Data Science, University of North Carolina at Charlotte, Charlotte, NC, United States; ^3^Department of Bioinformatics and Genomics, University of North Carolina at Charlotte, Charlotte, NC, United States; ^4^Department of Computer Science, University of North Carolina at Charlotte, Charlotte, NC, United States; ^5^Department of Geography and Earth Sciences, University of North Carolina at Charlotte, Charlotte, NC, United States

**Keywords:** COVID-19, epidemic, modeling, deep learning, multivariate

## Abstract

**Background:** Mathematical models are powerful tools to study COVID-19. However, one fundamental challenge in current modeling approaches is the lack of accurate and comprehensive data. Complex epidemiological systems such as COVID-19 are especially challenging to the commonly used mechanistic model when our understanding of this pandemic rapidly refreshes.

**Objective:** We aim to develop a data-driven workflow to extract, process, and develop deep learning (DL) methods to model the COVID-19 epidemic. We provide an alternative modeling approach to complement the current mechanistic modeling paradigm.

**Method:** We extensively searched, extracted, and annotated relevant datasets from over 60 official press releases in Hubei, China, in 2020. Multivariate long short-term memory (LSTM) models were developed with different architectures to track and predict multivariate COVID-19 time series for 1, 2, and 3 days ahead. As a comparison, univariate LSTMs were also developed to track new cases, total cases, and new deaths.

**Results:** A comprehensive dataset with 10 variables was retrieved and processed for 125 days in Hubei. Multivariate LSTM had reasonably good predictability on new deaths, hospitalization of both severe and critical patients, total discharges, and total monitored in hospital. Multivariate LSTM showed better results for new and total cases, and new deaths for 1-day-ahead prediction than univariate counterparts, but not for 2-day and 3-day-ahead predictions. Besides, more complex LSTM architecture seemed not to increase overall predictability in this study.

**Conclusion:** This study demonstrates the feasibility of DL models to complement current mechanistic approaches when the exact epidemiological mechanisms are still under investigation.

## Introduction

Mathematical models are important tools to understanding and predicting COVID-19 epidemic dynamics. A recent literature search in the NIH LitCovid online COVID-19 database revealed more than 6,000 peer-reviewed published modeling papers on the current pandemic (1). Among them, most models are in the mechanistic modeling paradigm. The most common mechanistic modeling approach is to construct a compartmental SEIR (Susceptible-Exposed-Infected-Recovery)-type model, fit the published case series to the model, and quantify key parameters such as basic reproduction number (*R*_0_) (2–4). Other less-common approaches include cross-scale modeling, agent-based modeling, and more recent advances in machine learning and deep learning (DL) models.

Mathematical models of epidemic dynamics, regardless of types, eventually all rely on the fundamental elements, the data, to operate. While there are adequate studies on various modeling approaches, relatively less emphasis has been put on the neglected yet critical data quality and reliability issues (5). Given the novelty of the SARS-CoV-2 pathogen, the definition of “cases” is not consistently and accurately defined across time and space. Case numbers are contingent on testing capacity and our knowledge about this novel pathogen, especially at the beginning the disease. COVID-19 has at least two distinct clinical stages: severe (which requires hospitalization including intensive care, use of ventilator) and non-severe (which usually does not involve intensive medical care) (6). These two stages have distinct consequences on the transition of the epidemiological state, especially from susceptible to infected, and from infected to recovery or death. Without the understanding and incorporation of these clinical insights, it is difficult to accurately model COVID-19 epidemic dynamics.

Many non-traditional data and metadata are available but are not well-explored for the complex socioepidemiological system of COVID-19 (5). In general, more data can help characterize complex systems with more resilience to input data bias. Previously neglected metadata provide additional insights in characterizing the unprecedented pandemic. However, these data are generally not well-organized, scattered across different places, and not in standardized reporting format (e.g., in a table or database).

**Figure 1:**
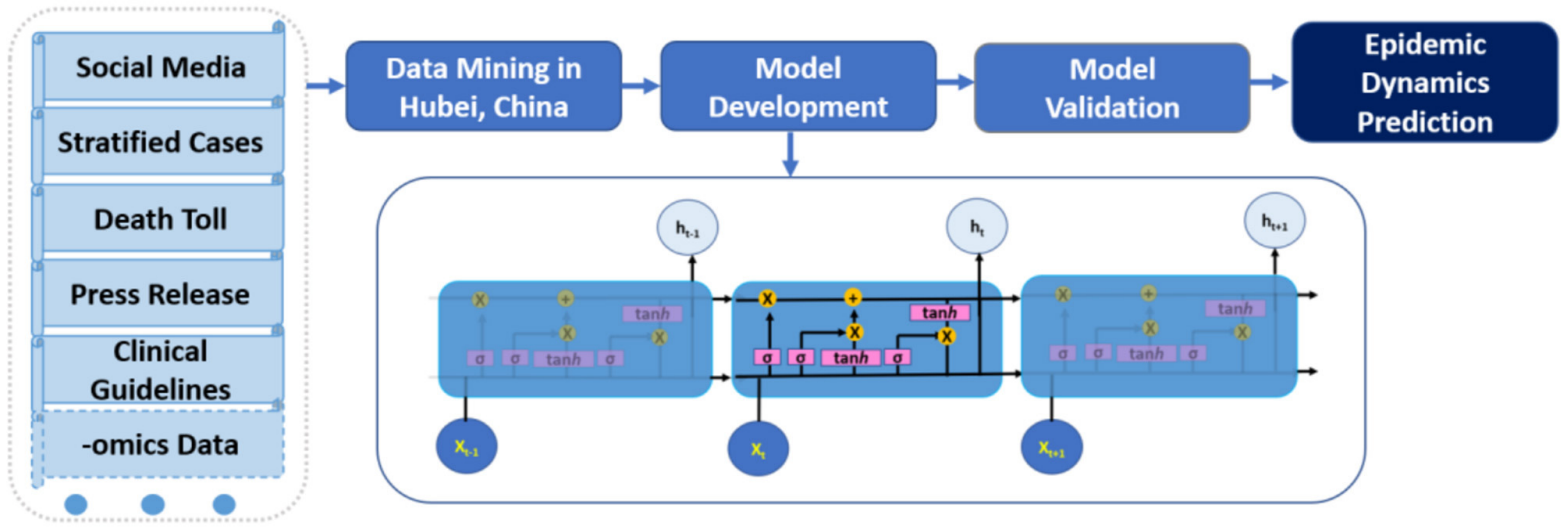
Schematic data mining and multivariate deep learning (long short-term memory LSTM) workflow for COVID-19 modeling.

DL models are based on deep neural networks which include convolution neural network (CNN), recurrent neural network (RNN), and generative adversarial network (GAN). Compared to non-data-driven methods, which usually focus on epidemiological mechanisms such as transmission and recovery, data-driven models are not driven by man-made assumptions about these mechanisms. Such assumptions may be misleading because a novel pandemic has many unknowns both clinically and epidemiologically. According to the Universal Approximation Theorem (7), even simple neural networks can approximate complex functions. Among various DL models, RNNs are particularly useful to handle time series data and have demonstrated their high performance in audio-visual analytics. Long short-term memory (LSTM) models, a type of RNN, have “remember” and “forget” gates, which are essential for LSTM to learn the high-level representation in time series data by adjusting how much information to keep (i.e., useful information) or forget (i.e., unuseful information) from previous time steps (Figure 1). After all, the fundamental goal of modeling infectious disease dynamics is to accurately represent the functional response of the epi-curve across time and space. Nevertheless, most current DL approaches on COVID-19 modeling are still univariate on reported case series (8–11), making them prone to the same data quality issue as other approaches.

In this study, we explore and demonstrate the feasibility of data-driven DL models, especially multivariate LSTM, on characterizing COVID-19 epidemic with additional metadata mining steps in Hubei, China.

## Methods

### Data Mining

Hubei Province, China, was selected in this study because it had a complete COVID-19 epidemic from starting to ending, a relatively large first wave of outbreak, and reasonable amount of information regarding the epidemic, although data quality was inconsistent at the beginning of the first wave of the epidemic. We checked the official Hubei Province COVID-19 press release from January 25, 2020 to May 15, 2020 (12). More than 60 publicly available government press releases were screened and archived. After the initial check, we designed a specific regular expression (regex) in Python to crawl the corresponding webpages and automatically extract information from each press release. We built a customized lookup table for the regex to identify specific data of interest, such as numbers of in-hospital monitoring, from the press release.

### Data Preprocessing

The raw data extracted from the press releases were not consistent. We retrospectively fit missing values with extrapolation, assuming the first case was on January 1, 2020 in this study as the first “suspected” case was reported around then. The last official press release was published on May 11, 2020, ~5 weeks after the lift of lockdown in Wuhan, Hubei, marking the end of the first wave of the epidemic.

After data preprocessing, all variables were fitted to the same length of 125d. To fit the DL model, the multivariate time series were required to be the same length. We applied a min-max scaler to transform continuous variable values to percentage and fed them into the DL workflow for increased efficiency. The percentage outputs from the DL model were then transformed back to continuous values to compare with actual observed values and evaluate model performance.

### Multivariate Deep-Learning Model Based on LSTM

The preprocessed multivariate time series were fed into the LSTM model. There were two types of multivariate LSTM settings; the first would use one variable (e.g., incident case numbers) as an output (i.e., dependent variable), while all other variables were used as predictors (i.e., independent/input variables). In this study, we used multivariate time series at the same time without differentiating the predictor (input) and response variable (output). The time series of the cumulative case, in theory, should have the same predictive power as the incident case, as the cumulative case is the sum of daily incident cases from the beginning to the current time step. However, the cumulative time series has less fluctuation than daily counts, as cumulatives were monotonically increasing. This property might have influences on LSTM performance.

LSTM requires the user to specify the number of past time steps and number of future steps to operate. These are known as “hyperparameters” in machine learning and DL models, and are user-definable values. In this study, we chose 3-day past time steps to predict 3 days ahead as a demonstration on how LSTM handled temporal autocorrelation. Shorter periods (e.g., 1 day) may ignore temporal patterns in the data, while longer periods (e.g., 7 days) may be too long for COVID-19 prognosis; therefore, 3 days were a reasonable hyperparameter of time step in LSTM. The dataset was then transformed in a series of 3-day moving windows, making the new dataset substantially larger than the original data with more information about the COVID-19 epidemic, especially potential temporal autocorrelation. This larger dataset was important for LSTM to learn high-level representation of the data and improve model performance.

LSTM architecture in this study included multiple stacked encoders and decoders. In theory, more complex architecture (i.e., more encoders and decoders) would reinforce the model to better learn the representation of the data (e.g., temporal pattern) and increase predictability, but might bear the risk of overfitting (9). We developed a simpler one-encoder one-decoder LSTM (E1D1) and a more complex two-encoder two-decoder LSTM (E2D2). We aimed to investigate whether more complex architecture E2D2 necessarily increase predictability in multivariate LSTM.

As a baseline comparison, we also developed individual univariate LSTM to predict incident cases, cumulative cases, and new deaths: the three mostly used variables in COVID-19 models. We compared model performance of these univariate LSTMs with results from multivariate LSTM. An illustration of the complete analytical workflow is shown in Figure 1. Detailed model architecture is provided in the Supplementary Material.

To run LSTM models, first 80% of the data was used to train LSTM. The trained model was then tested with the remaining 20% unseen data to evaluate model performance. Mean absolute error (MAE) was chosen for LSTM model performance evaluation by calculating the error between predicted value from the model and actual reported value in the data. Each variable derived from data mining and preprocessing steps would have its own MAE. The LSTM model was trained with 25 epochs, with the Huber loss function and Adam optimization method. We used Python 3.7 with additional Scikit Learn and Tensorflow 2.0 packages.

## Results

### Data Mining

After screening published COVID-19 situation reports and press releases, 10 variables were included in this study. These variables were: new confirmed cases, new deaths, new discharges from hospital, cumulative confirmed cases, cumulative hospitalization of COVID-19 patients in severe stage defined by the National Health Commission of China) (6), cumulative hospitalizations in critical stage, cumulative deaths, cumulative discharges, number of individuals tracked (i.e., contact tracing), and number of individuals with high likelihood of infection and being monitored in hospital (i.e., suspected cases). While case and death numbers were common variables in current COVID-19 models, we suggested that numbers of hospitalizations, discharges, tracing, and suspected cases could help depict the COVID-19 epidemic more comprehensively.

Additional data cleaning and extrapolation and interpolation were performed to make sure all time series of the 10 included variables had the same length of 125d, from January 1, 2020 to May 11, 2020. The dimension of the dataset was 125 by 10.

### Multivariate and Univariate LSTM

**Figure 2:**
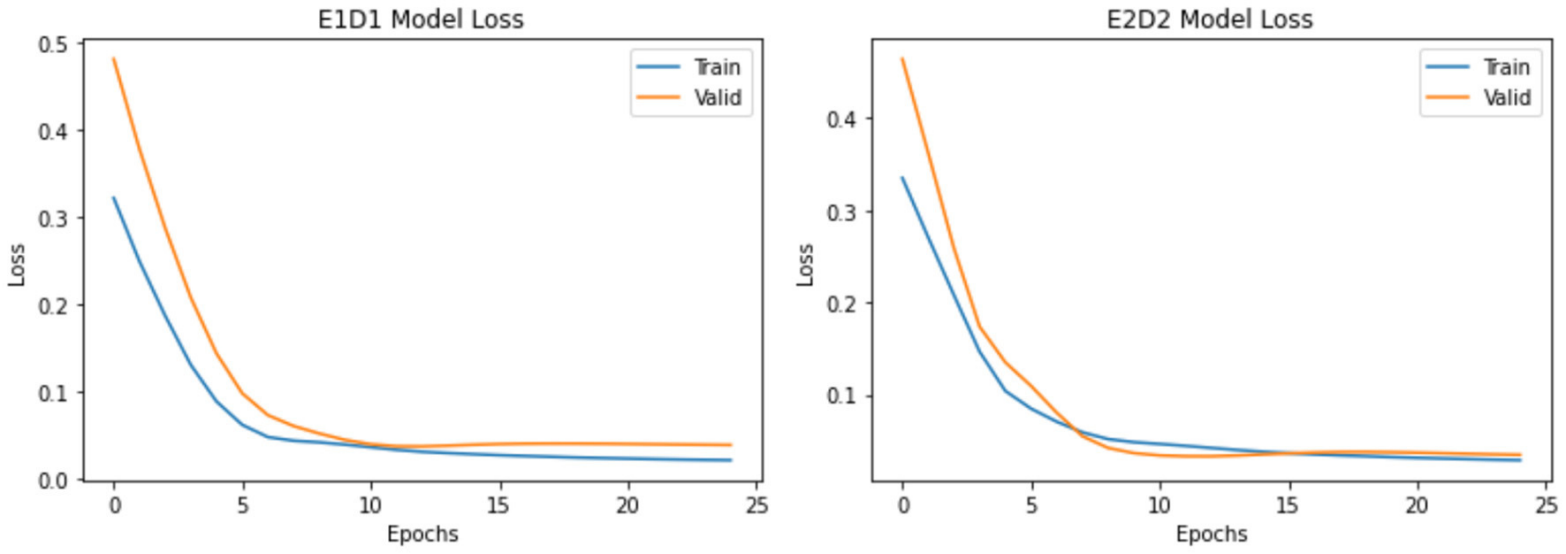
Comparison of model loss (multivariate E1D1 vs. E2D2).

Both LSTMs in E1D1 and E2D2 architectures (Figure 2 left and right panels, respectively) showed no evidence of overfitting or underfitting in either training (blue curve) or validation set (orange curve) in 25 epochs. Model losses diminished quickly after a few (<10) epochs in all scenarios. E2D2, the more complicated architecture, had a smaller difference between training and validation set, which was more desirable than E1D1.

The detailed MAEs of each LSTM architecture are provided in Table 1. For prediction of new cases number, more complicated E2D2 architecture provided better accuracy in 1-d ahead (687 vs. 919), but substantially worse in 2-d ahead (1,168 vs. 470), and again better prediction (1,039 vs. 1,383) in 3-d ahead prediction than simpler E1D1. For total cumulative cases, less complicated E1D1 architecture provided consistently better predictability than E2D2 across all three prediction steps (1, 2, and 3d). Because the Health Commission of China had changed the definition of “confirmed case” on February 2, 2020 to include clinical cases based on CT scan (6), and the case numbers had a large peak, which might not reflect the actual epidemic dynamics and proposed challenge for models to deal with, especially in the middle of a time series (i.e., “spikes”). Therefore, we also provided relative MAEs based on the maximum number of each variable (Table 1, values in parenthesis) to evaluate the model performance more comprehensively.

**Table 1:** Multivariate and Univariate Model Performance Comparison Between Different LSTM Architectures based on Mean Absolute Error (MAE).

**LSTM architecture**	**MAE E1D1**			**MAE E2D2**		
**Day/Variable**	**Day1**	**Day2**	**Day3**	**Day1**	**Day2**	**Day3**
New cases	919 (6.19)	470 (3.17)	1,383 (9.32)	687 (4.63)	1,168 (7.87)	1,039 (7.00)
Total cases	842 (1.24)	3,555 (5.22)	5,905 (8.67)	2,686 (3.94)	3,914 (5.74)	7,866 (11.54)
New deaths	11 (4.55)	8 (3.31)	18 (7.44)	0 (0.00)	15 (6.20)	20 (8.26)
New discharges	594 (23.11)	320 (12.45)	184 (7.16)	416 (16.19)	302 (11.75)	255 (9.92)
Hospital severe	670 (7.21)	215 (2.31)	84 (0.90)	669 (7.20)	299 (3.22)	144 (1.55)
Hospital critical	130 (5.22)	58 (2.33)	55 (2.21)	209 (8.39)	78 (3.13)	18 (0.72)
Total deaths	1,579 (35.00)	1,144 (25.35)	898 (19.90)	1,431 (31.72)	1,084 (24.02)	873 (19.35)
Total discharges	689 (1.07)	626 (0.97)	27 (0.04)	2,304 (3.57)	1,526 (2.37)	3,611 (5.60)
Total tracked	17,487 (6.17)	5,669 (2.00)	20,359 (7.19)	398 (0.14)	17,917 (6.33)	25,764 (9.10)
Total monitored	2,213 (2.85)	2,889 (3.72)	2,486 (3.20)	1,930 (2.48)	4,426 (5.70)	5,460 (7.03)
New cases univariate	1,235 (8.32)	63 (0.42)	394 (2.65)	877 (5.91)	190 (1.28)	60 (0.40)
Total cases univariate	5,720 (8.40)	705 (1.03)	3,640 (5.34)	2,814 (4.13)	1,042 (1.32)	1,920 (2.82)
New deaths univariate	21 (8.68)	2 (0.83)	5 (2.07)	14 (5.79)	5 (2.07)	4 (1.65)

*The first 10 variables were in the same multivariate LSTM model. The last three univariate LSTMs were different models based on their respective univariate inputs. Numbers in parentheses represented relative values as percentage*.

We further examined whether time series of other variables could be predicted more accurately because of higher input data reliability. Multivariate LSTM performed reasonably well in predicting new deaths, hospitalizations of patients in both severe and critical condition, as well as total number of discharges from the hospital. Once again, we did not observe that either E1D1 or E2D2 was superior to the other in all three prediction time steps. Therefore, multiple architectures might need to be developed for different tasks (e.g., immediate or short-term prediction). Total deaths and new discharges were having the worst predictability among the 10 included variables. While in theory total deaths and total discharges were the cumulative sum of daily deaths and daily discharges, data-driven LSTM seemed to treat daily and total numbers independently, thus resulting in vastly different predictability of these variables.

In addition, univariate LSTM performed substantially better in 2d- and 3d-ahead prediction of the incident case, total case, and new death than their multivariate LSTM counterparts, no matter what architecture was chosen (E1D1 or E2D2). Multivariate LSTM only outperformed univariate models in 1d-ahead prediction (Table 1) in both E1D1 and E2D2 architecture. Nevertheless, because of the inconsistency in “case” definition, the seemingly better predictability of univariate LSTM on case numbers should be interpreted with caution.

## Discussion

This DL approach is able to tackle some modeling challenges in current complex epidemiological systems such as the COVID-19 pandemic. In summary, not all variables had the same predictive power in the multivariate LSTM that we developed. New cases, total cases, new death, hospitalization of severe patients, hospitalization of critical condition patients, total discharged, total tracked, and total monitored in hospital had much better predictability than new discharge and total death. Univariate LSTM performed better than multivariate model in predicting both new case, total case, and new death for 2d- and 3d-ahead prediction, but multivariate LSTM outperformed at 1d-ahead prediction. We also tested 5d-ahead prediction, and the results were similar. In addition, more complex E2D2 architecture did not provide substantial performance boost over simpler E1D1 architecture. While the definitions of “case” were not consistent over time and space, we suggested that new death could be a more robust variable to track and predict during the COVID-19 epidemic. In general, there was no “one-size-fits-all” solution of LSTM architecture, and we suggest future case studies to develop several different architectures in parallel to identify the most appropriate one. In addition, LSTM is just one type of RNN besides other alternatives such as gated recurrent network (8).

LSTM is relatively easy to operate and straightforward to quickly adjust LSTM architecture by adding, removing, or revising existing layers, which are the building blocks for LSTM. More importantly, the multivariate LSTM developed in this study can be easily extended to further incorporate more data such as in the MIDAS GitHub repository (13). Other non-traditional data, such as social media, sensor-based data, and drone imaging, can be incorporated into the LSTM model to better characterize multiple aspects of COVID-19 dynamics (14–19). Decentralized blockchain techniques and robotics could also provide rich and secure inputs for data-driven models such as LSTM (19, 20).

Unlike mechanistic models, data-driven DL models generally do not require thorough understanding of disease mechanisms to work with, as DL is directly driven by underlying data. We suggest the data mining step is therefore essential: more data will increase model resilience against biases in case numbers (e.g., inconsistent case definition in early phase of COVID-19). Recent blockchain technology could facilitate data archiving and mitigate data tampering issues (19).

For DL, existing models developed in a certain region can be applied to other regions with distinct sociocultural backgrounds *via* the transfer learning technique (10). This is another key potency specific to DL models. Modeling and comparing epidemic dynamics across sociocultural backgrounds in different regions of the world is a challenging task to mechanistic models. Many sociocultural differences (e.g., public attitude toward interventions and willingness of compliance to these interventions) may drive COVID-19 dynamics differently, but the influence of these factors is difficult to quantify in mechanistic models. However, for neural-network-based DL models, we can fix existing network layers and “transfer” this pretrained model from one region to another with distinct sociocultural backgrounds.

There are some downsides of data-driven DL models. Most prominently, DL models generally do not have good interpretability, compared to explicit mechanistic models. Therefore, DL models generally cannot derive important parameters such as *R*_0_, which is the key in mechanistic models. We suggest that DL models are more appropriate for prediction than interpretation. The other technical challenge is that although DL modeling process has been substantially simplified with Keras and Tensorflow libraries, it still requires a substantial amount of programming experiences and skills. The modeling approach, process, and results could be opaque to stakeholders and concerned citizens.

## Conclusion

In this study, we describe a data-driven workflow on COVID-19 modeling, including data mining, cleaning, preprocessing, and DL with multivariate RNNs. We suggest that the multivariate LSTMs demonstrated in this study are not intended to replace the current mechanistic modeling approaches. DL models, when meticulously developed on robust datasets, are able to complement existing modeling approaches by providing a different angle on the complex epidemiological systems such as COVID-19.

## Data Availability Statement

The original contributions generated for this study are included in the article/Supplementary Material, further inquiries can be directed to the corresponding author/s.

## Author Contributions

TF performed original data mining and pre-processing. SC, KM, and RP developed deep learning model. RP, DJ, and J-CT supervised the study. SC wrote the manuscript. All authors discussed and approved the final manuscript for submission.

## Conflict of Interest

The authors declare that the research was conducted in the absence of any commercial or financial relationships that could be construed as a potential conflict of interest.
