# Supplementary material for: Exploring Feasibility of Multivariate Deep Learning Models in Predicting COVID-19 Epidemic
Source: Front Public Health. 2021 Jul 5;9:661615. doi: 10.3389/fpubh.2021.661615 (PMC8287417; doi:10.3389/fpubh.2021.661615)
Supplement: Supplementary file 1 [file Table_1.DOCX]

Supplementary Material

# Code

import pandas as pd

import numpy as np

from sklearn.preprocessing import MinMaxScaler

import matplotlib.pyplot as plt

import tensorflow as tf

import os

df=pd.read_csv('hubeidata.csv',header=0, low_memory=False,index_col=['date'])

#df.incident=df[["New_Death"]]

#df=df.incident

df.head()

# df.incident.head()

# df.incident is the univariate incident case series

# For this case, let's assume that

# Given past 3 days observation, forecast the next 3 days observations.

n_past = 3

n_future = 3

n_features = 10

# change n_features = 1 for univariate LSTM otherwise 10 for multivariate

train_df,test_df = df[1:100], df[101:]  # 80% and 20%

train_df.shape,test_df.shape

train = train_df

scalers={}

for i in train_df.columns:

    scaler = MinMaxScaler(feature_range=(-1,1))

    s_s = scaler.fit_transform(train[i].values.reshape(-1,1))

    s_s=np.reshape(s_s,len(s_s))

    scalers['scaler_'+ i] = scaler

    train[i]=s_s

test = test_df

for i in train_df.columns:

    scaler = scalers['scaler_'+i]

    s_s = scaler.transform(test[i].values.reshape(-1,1))

    s_s=np.reshape(s_s,len(s_s))

    scalers['scaler_'+i] = scaler

    test[i]=s_s

def split_series(series, n_past, n_future):

  #

  # n_past ==> no of past observations

  #

  # n_future ==> no of future observations

  #

  X, y = list(), list()

  for window_start in range(len(series)):

    past_end = window_start + n_past

    future_end = past_end + n_future

    if future_end > len(series):

      break

    # slicing the past and future parts of the window

    past, future = series[window_start:past_end, :], series[past_end:future_end, :]

    X.append(past)

    y.append(future)

  return np.array(X), np.array(y)

# E1D1

# n_features ==> no of features at each timestep in the data.

#

encoder_inputs = tf.keras.layers.Input(shape=(n_past, n_features))

encoder_l1 = tf.keras.layers.LSTM(100, return_state=True)

encoder_outputs1 = encoder_l1(encoder_inputs)

encoder_states1 = encoder_outputs1[1:]

#

decoder_inputs = tf.keras.layers.RepeatVector(n_future)(encoder_outputs1[0])

#

decoder_l1 = tf.keras.layers.LSTM(100, return_sequences=True)(decoder_inputs,initial_state = encoder_states1)

decoder_outputs1 = tf.keras.layers.TimeDistributed(tf.keras.layers.Dense(n_features))(decoder_l1)

#

model_e1d1 = tf.keras.models.Model(encoder_inputs,decoder_outputs1)

#

model_e1d1.summary()

# E2D2

# n_features ==> no of features at each timestep in the data.

#

encoder_inputs = tf.keras.layers.Input(shape=(n_past, n_features))

encoder_l1 = tf.keras.layers.LSTM(100,return_sequences = True, return_state=True)

encoder_outputs1 = encoder_l1(encoder_inputs)

encoder_states1 = encoder_outputs1[1:]

encoder_l2 = tf.keras.layers.LSTM(100, return_state=True)

encoder_outputs2 = encoder_l2(encoder_outputs1[0])

encoder_states2 = encoder_outputs2[1:]

#

decoder_inputs = tf.keras.layers.RepeatVector(n_future)(encoder_outputs2[0])

#

decoder_l1 = tf.keras.layers.LSTM(100, return_sequences=True)(decoder_inputs,initial_state = encoder_states1)

decoder_l2 = tf.keras.layers.LSTM(100, return_sequences=True)(decoder_l1,initial_state = encoder_states2)

decoder_outputs2 = tf.keras.layers.TimeDistributed(tf.keras.layers.Dense(n_features))(decoder_l2)

#

model_e2d2 = tf.keras.models.Model(encoder_inputs,decoder_outputs2)

#

model_e2d2.summary()

reduce_lr = tf.keras.callbacks.LearningRateScheduler(lambda x: 1e-3 * 0.90 ** x)

model_e1d1.compile(optimizer=tf.keras.optimizers.Adam(), loss=tf.keras.losses.Huber())

history_e1d1=model_e1d1.fit(X_train,y_train,epochs=25,validation_data=(X_test,y_test),batch_size=32,verbose=0,callbacks=[reduce_lr])

model_e2d2.compile(optimizer=tf.keras.optimizers.Adam(), loss=tf.keras.losses.Huber())

history_e2d2=model_e2d2.fit(X_train,y_train,epochs=25,validation_data=(X_test,y_test),batch_size=32,verbose=0,callbacks=[reduce_lr])

plt.plot(history_e1d1.history['loss'])

plt.plot(history_e1d1.history['val_loss'])

plt.title("E1D1 Model Loss")

plt.xlabel('Epochs')

plt.ylabel('Loss')

plt.legend(['Train', 'Valid'])

plt.show()

plt.plot(history_e2d2.history['loss'])

plt.plot(history_e2d2.history['val_loss'])

plt.title("E2D2 Model Loss")

plt.xlabel('Epochs')

plt.ylabel('Loss')

plt.legend(['Train', 'Valid'])

plt.show()

pred1_e1d1=model_e1d1.predict(X_test)

pred1_e2d2=model_e2d2.predict(X_test)

pred_e1d1=model_e1d1.predict(X_train)

pred_e2d2=model_e2d2.predict(X_train)

for index,i in enumerate(train_df.columns):

    scaler = scalers['scaler_'+i]

    pred1_e1d1[:,:,index]=scaler.inverse_transform(pred1_e1d1[:,:,index])

    pred_e1d1[:,:,index]=scaler.inverse_transform(pred_e1d1[:,:,index])

    pred1_e2d2[:,:,index]=scaler.inverse_transform(pred1_e2d2[:,:,index])

    pred_e2d2[:,:,index]=scaler.inverse_transform(pred_e2d2[:,:,index])

    y_train[:,:,index]=scaler.inverse_transform(y_train[:,:,index])

    y_test[:,:,index]=scaler.inverse_transform(y_test[:,:,index])

from sklearn.metrics import mean_absolute_error

for index,i in enumerate(train_df.columns):

  print(i)

  for j in range(1,4):

    # range from 1 to days of prediction +1

    print("Day ",j,":")

    print("MAE-E1D1 : ",mean_absolute_error(y_test[:,j-1,index],pred1_e1d1[:,j-1,index]),end=", ")

    print("MAE-E2D2 : ",mean_absolute_error(y_test[:,j-1,index],pred1_e2d2[:,j-1,index]))

  print()

  print()

| date | New_Confirmed | New_Death | New_Discharged | Total_Confirmed | Total_Hospitalization_Severe | Total_Hospitalization_Critical | Total_Death | Total_Discharged | Total_Track | Total_Observe |
| --- | --- | --- | --- | --- | --- | --- | --- | --- | --- | --- |
| 101 | 0 | 0 | 0 | 0 | 0 | 0 | 0 | 0 | 0 | 0 |
| 102 | 0 | 0 | 0 | 0 | 0 | 0 | 0 | 0 | 0 | 0 |
| 103 | 0 | 0 | 0 | 0 | 0 | 0 | 0 | 0 | 0 | 0 |
| 104 | 0 | 0 | 0 | 0 | 0 | 0 | 0 | 0 | 0 | 0 |
| 105 | 0 | 0 | 0 | 0 | 0 | 0 | 0 | 0 | 0 | 0 |
| 106 | 0 | 0 | 0 | 0 | 0 | 0 | 0 | 0 | 0 | 0 |
| 107 | 0 | 0 | 0 | 0 | 0 | 0 | 0 | 0 | 0 | 0 |
| 108 | 0 | 0 | 0 | 0 | 0 | 0 | 0 | 0 | 0 | 0 |
| 109 | 41 | 0 | 0 | 0 | 41 | 0 | 0 | 0 | 0 | 0 |
| 110 | 0 | 1 | 0 | 41 | 41 | 15 | 1 | 0 | 0 | 0 |
| 111 | 0 | 0 | 0 | 41 | 41 | 15 | 1 | 0 | 0 | 0 |
| 112 | 0 | 0 | 0 | 41 | 41 | 15 | 1 | 3 | 0 | 0 |
| 113 | 0 | 0 | 0 | 41 | 41 | 15 | 1 | 6 | 0 | 0 |
| 114 | 0 | 0 | 0 | 41 | 41 | 15 | 1 | 9 | 0 | 0 |
| 115 | 0 | 1 | 0 | 41 | 41 | 15 | 2 | 12 | 0 | 0 |
| 116 | 4 | 0 | 0 | 41 | 41 | 15 | 2 | 15 | 0 | 0 |
| 117 | 17 | 0 | 0 | 45 | 41 | 15 | 2 | 18 | 0 | 0 |
| 118 | 59 | 1 | 0 | 62 | 41 | 15 | 3 | 21 | 782 | 0 |
| 119 | 77 | 0 | 0 | 121 | 41 | 15 | 3 | 24 | 2942 | 0 |
| 120 | 93 | 3 | 0 | 198 | 42 | 15 | 6 | 27 | 3662 | 0 |
| 121 | 149 | 3 | 0 | 291 | 44 | 15 | 9 | 30 | 4382 | 0 |
| 122 | 131 | 8 | 0 | 440 | 49 | 16 | 17 | 33 | 4622 | 0 |
| 123 | 259 | 8 | 0 | 571 | 60 | 19 | 25 | 36 | 5103 | 307 |
| 124 | 222 | 16 | 0 | 830 | 83 | 26 | 41 | 39 | 6065 | 2506 |
| 125 | 323 | 13 | 10 | 1052 | 129 | 40 | 52 | 42 | 7989 | 6904 |
| 126 | 371 | 24 | 2 | 1423 | 221 | 69 | 76 | 44 | 10394 | 9103 |
| 127 | 1291 | 24 | 0 | 2714 | 563 | 127 | 100 | 47 | 16904 | 15559 |
| 128 | 840 | 25 | 0 | 3554 | 671 | 228 | 125 | 80 | 22095 | 20366 |
| 129 | 1032 | 37 | 10 | 4586 | 711 | 277 | 162 | 90 | 28780 | 26632 |
| 130 | 1220 | 42 | 26 | 5806 | 804 | 290 | 204 | 116 | 35144 | 32340 |
| 131 | 1347 | 45 | 50 | 7153 | 956 | 338 | 249 | 166 | 41075 | 36838 |
| 201 | 1921 | 45 | 49 | 9074 | 1118 | 444 | 294 | 215 | 48571 | 43121 |
| 202 | 2103 | 56 | 80 | 11177 | 1223 | 478 | 350 | 295 | 56088 | 48171 |
| 203 | 2345 | 64 | 101 | 13522 | 1567 | 576 | 414 | 396 | 68988 | 58544 |
| 204 | 3156 | 65 | 125 | 16678 | 1809 | 711 | 479 | 520 | 81039 | 66764 |
| 205 | 2987 | 70 | 113 | 19665 | 2328 | 756 | 549 | 633 | 90997 | 64127 |
| 206 | 2447 | 69 | 184 | 22112 | 3161 | 841 | 618 | 817 | 101599 | 64057 |
| 207 | 2841 | 81 | 298 | 24953 | 4188 | 1007 | 699 | 1115 | 114044 | 67802 |
| 208 | 2147 | 81 | 324 | 27100 | 4093 | 1154 | 780 | 1439 | 123827 | 70438 |
| 209 | 2618 | 91 | 356 | 29631 | 4269 | 1236 | 871 | 1795 | 132555 | 73127 |
| 210 | 2097 | 103 | 427 | 31728 | 5046 | 1298 | 974 | 2222 | 144279 | 76207 |
| 211 | 1638 | 94 | 417 | 33366 | 5724 | 1517 | 1068 | 2639 | 152251 | 77195 |
| 212 | 14840 | 242 | 802 | 48206 | 5647 | 1437 | 1310 | 3441 | 158377 | 77308 |
| 213 | 4823 | 116 | 690 | 51986 | 7593 | 1685 | 1310 | 3441 | 166818 | 77685 |
| 214 | 2420 | 139 | 912 | 54406 | 8276 | 1876 | 1457 | 4774 | 176148 | 77323 |
| 215 | 1843 | 139 | 849 | 56249 | 8439 | 1957 | 1596 | 5623 | 183183 | 74261 |
| 216 | 1933 | 100 | 1016 | 58182 | 8024 | 1773 | 1696 | 6639 | 191434 | 71613 |
| 217 | 1807 | 93 | 1223 | 59989 | 9117 | 1853 | 1789 | 7862 | 199322 | 69270 |
| 218 | 1693 | 132 | 1266 | 61682 | 9289 | 1957 | 1921 | 9128 | 206087 | 68345 |
| 219 | 349 | 108 | 1209 | 62031 | 9128 | 2050 | 2029 | 10337 | 214093 | 65525 |
| 221 | 366 | 106 | 1767 | 63454 | 8400 | 2492 | 2250 | 13557 | 234217 | 62787 |
| 222 | 630 | 96 | 1742 | 64084 | 8583 | 1845 | 2346 | 15299 | 240937 | 61181 |
| 223 | 398 | 149 | 1439 | 64287 | 7776 | 1654 | 2495 | 16738 | 246781 | 58575 |
| 225 | 401 | 52 | 2058 | 65187 | 6840 | 1486 | 2615 | 20912 | 255750 | 49438 |
| 228 | 423 | 45 | 2492 | 66337 | 6056 | 1314 | 2727 | 28895 | 263916 | 39303 |
| 229 | 570 | 34 | 2292 | 66907 | 5858 | 1249 | 2761 | 31187 | 265617 | 35766 |
| 301 | 196 | 42 | 2570 | 67103 | 5646 | 1226 | 2803 | 33757 | 267585 | 32143 |
| 304 | 134 | 31 | 1923 | 67466 | 4747 | 1041 | 2902 | 40479 | 270593 | 22801 |
| 305 | 126 | 29 | 1487 | 67592 | 4592 | 996 | 2931 | 41966 | 271959 | 21063 |
| 306 | 74 | 28 | 1502 | 67666 | 4395 | 964 | 2959 | 43468 | 272837 | 18296 |
| 308 | 36 | 21 | 1422 | 67743 | 4101 | 890 | 3007 | 46433 | 273617 | 13211 |
| 309 | 17 | 17 | 1152 | 67760 | 3855 | 846 | 3024 | 47585 | 273921 | 10832 |
| 310 | 13 | 22 | 1471 | 67773 | 3613 | 799 | 3046 | 49056 | 274109 | 9066 |
| 311 | 8 | 10 | 1242 | 67781 | 3453 | 727 | 3056 | 50298 | 274790 | 7964 |
| 312 | 5 | 6 | 1255 | 67786 | 3251 | 697 | 3062 | 51553 | 274992 | 6810 |
| 313 | 4 | 13 | 1390 | 67790 | 2896 | 647 | 3075 | 52943 | 275224 | 5887 |
| 314 | 4 | 10 | 1335 | 67794 | 2551 | 612 | 3085 | 54278 | 275362 | 5281 |
| 315 | 4 | 14 | 816 | 67798 | 2403 | 572 | 3099 | 55094 | 275503 | 4699 |
| 316 | 1 | 12 | 893 | 67799 | 2243 | 539 | 3111 | 55987 | 275696 | 4122 |
| 317 | 1 | 11 | 896 | 67800 | 2077 | 503 | 3122 | 56883 | 275817 | 3866 |
| 318 | 0 | 8 | 795 | 67800 | 1809 | 465 | 3130 | 57678 | 275883 | 3463 |
| 319 | 0 | 2 | 703 | 67800 | 1657 | 441 | 3132 | 58381 | 275955 | 3011 |
| 320 | 0 | 7 | 561 | 67800 | 1521 | 406 | 3139 | 58942 | 276026 | 2433 |
| 321 | 0 | 5 | 490 | 67800 | 1419 | 393 | 3144 | 59432 | 276153 | 1771 |
| 322 | 0 | 9 | 447 | 67800 | 1343 | 371 | 3153 | 59879 | 276227 | 1526 |
| 323 | 1 | 7 | 444 | 67801 | 1203 | 336 | 3160 | 60323 | 276597 | 1491 |
| 324 | 0 | 3 | 487 | 67801 | 1050 | 318 | 3163 | 60810 | 276909 | 1467 |
| 325 | 0 | 6 | 391 | 67801 | 914 | 287 | 3169 | 61201 | 277066 | 1344 |
| 326 | 0 | 5 | 530 | 67801 | 754 | 245 | 3174 | 61731 | 277213 | 1329 |
| 327 | 0 | 3 | 367 | 67801 | 659 | 227 | 3177 | 62098 | 277271 | 1352 |
| 328 | 0 | 5 | 467 | 67801 | 515 | 195 | 3182 | 62565 | 277367 | 1262 |
| 329 | 0 | 4 | 317 | 67801 | 427 | 174 | 3186 | 62882 | 277963 | 1250 |
| 330 | 0 | 1 | 271 | 67801 | 350 | 147 | 3187 | 63153 | 278089 | 1217 |
| 331 | 1 | 6 | 173 | 67802 | 311 | 124 | 3193 | 63326 | 278179 | 1230 |
| 401 | 0 | 6 | 145 | 67802 | 280 | 120 | 3199 | 63471 | 278370 | 1222 |
| 402 | 0 | 4 | 141 | 67802 | 239 | 110 | 3203 | 63612 | 278596 | 1386 |
| 403 | 1 | 4 | 150 | 67803 | 194 | 109 | 3207 | 63762 | 278797 | 1436 |
| 404 | 0 | 3 | 183 | 67803 | 171 | 96 | 3210 | 63945 | 279059 | 1607 |
| 405 | 0 | 1 | 69 | 67803 | 149 | 85 | 3212 | 64014 | 279236 | 1601 |
| 406 | 0 | 0 | 59 | 67803 | 111 | 73 | 3212 | 64073 | 279385 | 1611 |
| 407 | 0 | 1 | 69 | 67803 | 84 | 74 | 3213 | 64142 | 279553 | 1658 |
| 408 | 0 | 2 | 45 | 67803 | 70 | 67 | 3215 | 64187 | 279668 | 1650 |
| 409 | 0 | 1 | 49 | 67803 | 54 | 49 | 3216 | 64236 | 279767 | 1634 |
| 410 | 0 | 3 | 28 | 67803 | 51 | 44 | 3219 | 64264 | 279940 | 1703 |
| 411 | 0 | 0 | 17 | 67803 | 50 | 43 | 3219 | 64281 | 280029 | 1722 |
| 412 | 0 | 2 | 57 | 67803 | 38 | 37 | 3221 | 64338 | 280200 | 1815 |
| 413 | 0 | 0 | 25 | 67803 | 32 | 37 | 3221 | 64363 | 280307 | 1785 |
| 414 | 0 | 1 | 39 | 67803 | 24 | 33 | 3222 | 64402 | 280458 | 1868 |
| 415 | 0 | 0 | 33 | 67803 | 18 | 20 | 3222 | 64435 | 280675 | 1934 |
| 416 | 0 | 0 | 17 | 67803 | 15 | 17 | 3222 | 64452 | 280847 | 1994 |
| 417 | 0 | 0 | 7 | 68128 | 9 | 18 | 4512 | 63494 | 281022 | 1997 |
| 418 | 0 | 0 | 13 | 68128 | 6 | 16 | 4512 | 63507 | 281141 | 1974 |
| 419 | 0 | 0 | 4 | 68128 | 6 | 14 | 4512 | 63511 | 281229 | 1906 |
| 420 | 0 | 0 | 3 | 68128 | 6 | 13 | 4512 | 63514 | 281438 | 1962 |
| 421 | 0 | 0 | 5 | 68128 | 3 | 12 | 4512 | 63519 | 281530 | 1893 |
| 422 | 0 | 0 | 28 | 68128 | 1 | 1 | 4512 | 63547 | 281620 | 1812 |
| 423 | 0 | 0 | 22 | 68128 | 1 | 0 | 4512 | 63569 | 281721 | 1723 |
| 424 | 0 | 0 | 24 | 68128 | 0 | 0 | 4512 | 63593 | 281874 | 1712 |
| 425 | 0 | 0 | 11 | 68128 | 0 | 0 | 4512 | 63604 | 281984 | 1706 |
| 426 | 0 | 0 | 12 | 68128 | 0 | 0 | 4512 | 63616 | 282107 | 1728 |
| 427 | 0 | 0 | 0 | 68128 | 0 | 0 | 4512 | 63616 | 282236 | 1696 |
| 428 | 0 | 0 | 0 | 68128 | 0 | 0 | 4512 | 63616 | 282324 | 1675 |
| 429 | 0 | 0 | 0 | 68128 | 0 | 0 | 4512 | 63616 | 282382 | 1517 |
| 430 | 0 | 0 | 0 | 68128 | 0 | 0 | 4512 | 63616 | 282482 | 1434 |
| 501 | 0 | 0 | 0 | 68128 | 0 | 0 | 4512 | 63616 | 282546 | 1378 |
| 502 | 0 | 0 | 0 | 68128 | 0 | 0 | 4512 | 63616 | 282632 | 1383 |
| 503 | 0 | 0 | 0 | 68128 | 0 | 0 | 4512 | 63616 | 282701 | 1280 |
| 504 | 0 | 0 | 0 | 68128 | 0 | 0 | 4512 | 63616 | 282751 | 1209 |
| 505 | 0 | 0 | 0 | 68128 | 0 | 0 | 4512 | 63616 | 282812 | 1192 |
| 506 | 0 | 0 | 0 | 68128 | 0 | 0 | 4512 | 63616 | 282865 | 1118 |
| 507 | 0 | 0 | 0 | 68128 | 0 | 0 | 4512 | 63616 | 282926 | 1072 |
| 508 | 0 | 0 | 0 | 68128 | 0 | 0 | 4512 | 63616 | 283009 | 1053 |
| 509 | 1 | 0 | 0 | 68129 | 0 | 0 | 4512 | 63616 | 283074 | 1001 |
| 510 | 5 | 0 | 0 | 68134 | 0 | 0 | 4512 | 63616 | 283118 | 984 |
| 511 | 0 | 0 | 0 | 68134 | 0 | 1 | 4512 | 63616 | 283216 | 983 |
